# Multidisciplinary Management of Complicated Crown-Root Fracture of an Anterior Tooth Undergoing Apexification

**DOI:** 10.1155/2015/521013

**Published:** 2015-06-04

**Authors:** Merve Mese, Merve Akcay, Bilal Yasa, Huseyin Akcay

**Affiliations:** ^1^Department of Pedodontics, Faculty of Dentistry, University of Izmir Katip Celebi, 35640 Izmir, Turkey; ^2^Department of Restorative Dentistry, Faculty of Dentistry, University of Izmir Katip Celebi, 35640 Izmir, Turkey; ^3^Department of Oral and Maxillofacial Surgery, Faculty of Dentistry, University of Izmir Katip Celebi, 35640 Izmir, Turkey

## Abstract

The purpose of this case report was to present the multidisciplinary management of a subgingival crown-root fracture of a patient undergoing apexification treatment. A 12-year-old male patient was referred to the pediatric dentistry clinic with an extensive tooth fracture of the right permanent maxillary lateral incisor. Clinical and radiographic examinations revealed the presence of a complicated crown-root fracture, which had elongated to the buccal subgingival area. The dental history disclosed that the apexification procedure had been started to be performed after his first trauma experience and he had neglected his appointment. The coronal fragment was gently extracted; endodontic treatment was performed; flap surgery was performed to make the fracture line visible. The coronal fragment was reattached to the root fragment with a dual-cure luting composite. A fiber post was stabilized and the access cavity of the tooth was restored with composite resin. At the end of the 24th month, the tooth was asymptomatic, functionally, esthetically acceptable and had no periapical pathology. It is important for the patients undergoing apexification treatment to keep their appointments because of the fracture risk. Restoration of the fractured tooth by preparing retention grooves and a bonding fiber-reinforced post are effective and necessary approaches for successful management.

## 1. Introduction

The treatment of a pulpal injury of immature teeth creates technical difficulties for endodontic treatment, and a root-end closure (apexification) procedure is usually required to induce a calcified barrier in an open apex. Patients undergoing apexifications treatment have high crown-root fracture risks because of the thin dentinal walls [1, 2].

A permanent tooth suffering from trauma could be a considerable problem especially for young patients owing to functional and esthetic causes [3, 4]. A complicated crown-root fracture is a type of traumatic dental injury that involves the enamel, dentin, and cementum. This type of fracture usually results from a horizontal impact [5]. Crown-root fractures may be classified as complicated, due to pulpal involvement, which are more frequent, or noncomplicated, which have an absence of pulpal involvement [6, 7]. Crown-root fractures are quite common and frequently present treatment problems due to the complex nature of the injury [8]. To perform optimal treatment in such cases it is necessary to use the combined efforts of an interdisciplinary approach, which involves representatives from pediatric dentistry, endodontics, oral surgery, orthodontics, and restorative dentistry [9, 10].

The aim of this case report was to present the multidisciplinary management of a complicated crown-root fracture that extended subgingivally in an anterior tooth.

## 2. Case Report

A 12-year-old male patient was referred to the pediatric dentistry clinic at the University of Izmir Katip Celebi Faculty of Dentistry complaining of a tooth fracture after biting into chocolate.

**Figure 1:**
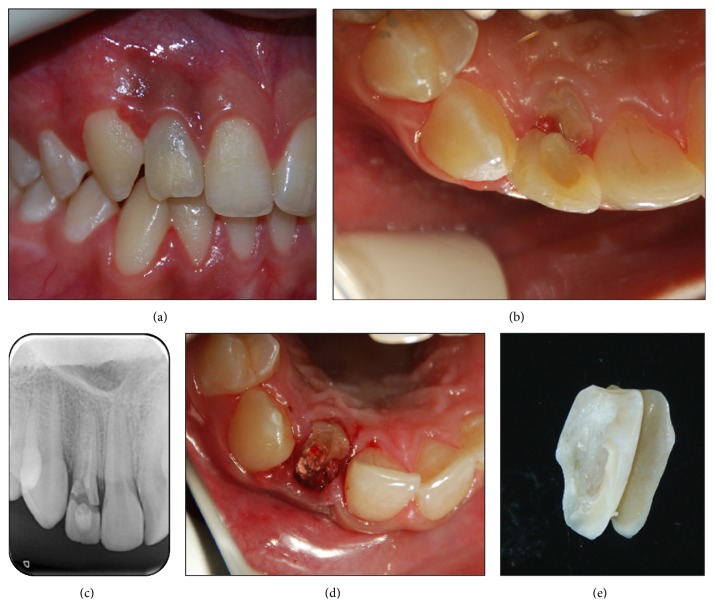
(a) Intraoral buccal view of a crown-root fracture. (b) Intraoral occlusal view of a crown-root fracture. (c) Preoperative intraoral radiograph showing the extension of the fracture. (d) View of the remaining root fragment after the removal of the coronal fragment. (e) View of the extracted coronal fragment.

The clinical examination revealed a complicated crown-root fracture of the right permanent maxillary lateral incisor with a mobile coronal tooth fragment that extended subgingivally in the buccal area (Figures 1(a)-1(b)). A radiographic examination showed that residual calcium hydroxide paste existed in the root canal and the fractured tooth had a mature apex (Figure 1(c)). The patient's medical history was noncontributory. The patient's dental history disclosed that the patient had suffered dental trauma without any fracture nearly 18 months earlier. An apexification procedure had started to be performed at that time, but the patient neglected his appointment for about 12 months. Because of the thin and fragile root wall, the tooth was fractured 11 months after his last appointment.

A temporary restoration was present on the coronal tooth fragment and part of the coronal fragment was mobile and attached to the gingiva. Under local anesthesia, the coronal fragment was gently extracted (Figures 1(d)-1(e)). Because of the completion of calcification of the apical root area, endodontic treatment was planned during the same appointment.

**Figure 2:**
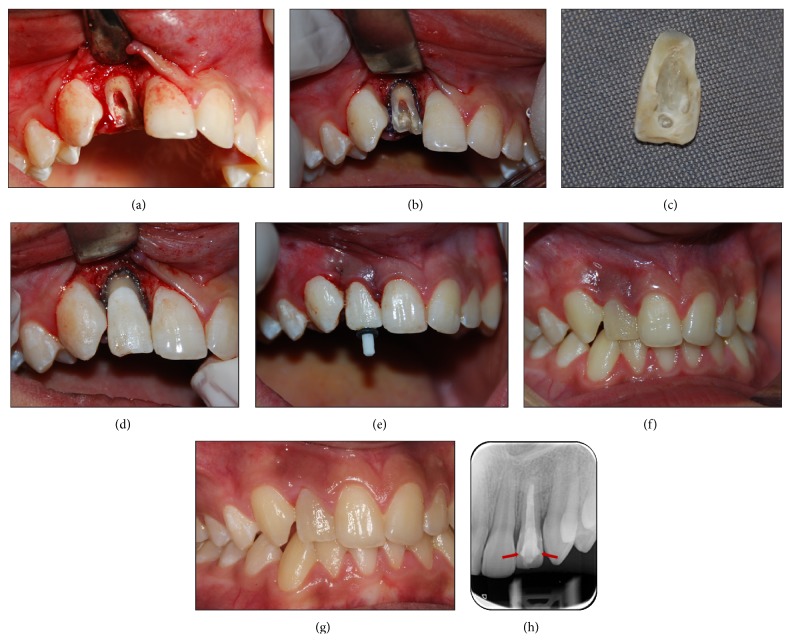
(a) Exposing the fracture line surgically. (b) Retraction cord application. (c) Grooves created on the coronal fragment. (d) Reattachment of the coronal fragment with a dual-cure luting composite. (e) Checking the adaptation of the resin fiber post into the root canal. (f) Intraoral view of the 1-week follow-up exam after reattachment. (g) Intraoral view of the 24-month follow-up exam after reattachment. (h) Periapical radiograph of the 24-month follow-up exam showing no pathologic alteration. Notice the grooves in the coronal fragment (arrows).

After preparing an endodontic access cavity, the root canal working length was determined to be 20 mm with a periapical radiograph. Due to the overcalcification of the apical root area, the root canal length was determined to be 2 mm shorter. Using endodontic K-files and H-files, shaping and cleaning of the root canal were performed. After the canal preparation, gutta-percha cones (Meta-Biomed, Cheongwon, Korea) were then lightly coated with an epoxy resin-based sealer (AH Plus Jet, Dentsply DeTrey, Konstanz, Germany) and the root canal treatment was completed with a lateral condensation. Subsequent to the canal obturation, a flap surgery was performed to make the fractured root face visible (Figure 2(a)). Hemorrhage was reduced by the application of an adrenaline-soaked gauze with pressure. A retraction cord impregnated with 25% aluminum chloride retraction solution (Viscostat Clear, Ultradent, South Jordan, UT, USA) was placed on the fractured tooth borderline to control the sulcular hemorrhage (Figure 2(b)).

At the same time, retention grooves were created on the coronal fragment with a round diamond bur (Figure 2(c)), and acid etching (Vococid, Voco, Cuxhaven, Germany) was applied to the enamel of the coronal fragment. After hemorrhage control, a thin adhesive system (Clearfil SE Bond, Kuraray, Osaka, Japan) was applied to both the coronal and root fragments. By using a dual-curing luting composite (Variolink N, Ivoclar, Vivadent, Schaan, Liechtenstein), the coronal fragment was reattached to the root fragment and then polymerized (Figure 2(d)). The flap was then reapproximated to its original position and esthetically sutured (Figure 2(e)).

The root canal obturation was removed with drills to place a fiber post into the root canal for supporting the coronal fragment. The post space was etched and an adhesive system (Syntac, Ivoclar) of luting composite was applied. We then performed a 9% hydrofluoric acid etching and silanization (Porcelain Etch and Silane, Ultradent, South Jordan, UT, USA) of the post, which was a size #2 glass fiber post with a 1.4 mm diameter (Cytec Blanco, Hahnenkratt, Königsbach-Stein, Germany). The post was placed at the length of 11 mm and was stabilized into the canal with a dual-curing luting composite (Variolink N, Ivoclar) (Figure 2(e)). After cutting the excessive post, access to the tooth was restored with a composite resin (Clearfil Majesty, Kuraray, Okayama, Japan). After finishing and polishing, the restoration was checked radiographically for occlusal interferences. Oral hygiene instructions were given to the patient and a soft food diet was advised.

The patient was recalled at 1 week, 1 month, 12 months, and 24 months after the treatment for follow-up examination. At the end of the follow-up visits, the clinical and radiological investigation of the tooth revealed that the tooth was asymptomatic, functional, and esthetically acceptable with no evident clinical signs and no periapical pathology (Figures 2(f)–2(h)).

## 3. Discussion

Apexification is a reasonable treatment for teeth with open apices and nonvital pulp. Calcium hydroxide apexification remains the most widely used technique for treatment of these teeth. However, the traditional use of calcium hydroxide to accomplish apexification has some disadvantages: (i) there is a requirement for multiple visits over a lengthy time period (average 12 months) [11] and (ii) the calcium hydroxide has been shown both in clinical studies [1, 12] and in experimental studies [13, 14] to lead to a weakening of the root structure, often resulting in cervical root fractures [15]. In the present study, the management of a fracture complication of an anterior tooth undergoing apexification was presented.

For cases of complicated crown-root fractures, there are several proposed treatment options including a mucogingival flap, an osteotomy/osteoplasty, and orthodontic or surgical extrusion followed by the reattachment of the original fragment. Another option includes the restoration of the tooth crown with a restorative material or prosthetic rehabilitation of the tooth depending on the location of the fracture line [16–20]. If the fracture fragment is available, reattachment should be the first choice of treatment [21, 22]. As described in previous studies [23–25], this case report shows that the adhesive reattachment of the original fragment offers a conservative, esthetic, and cost-effective restorative option. Furthermore, it is an acceptable alternative to resin-based composite restorations for restoring esthetics and function of obliquely fractured teeth. Reattachment of an autogenous tooth fragment also has the advantage of biological width, which is the sum of the epithelial and connective tissue attachment lengths [26].

Some reattachment techniques, such as direct reattachment of the fragment, internal enamel groove, internal dentinal groove, and external enamel groove in a V-shape, have been used with better results when compared with the direct reattachment of the fragments [27–29]. Previous studies indicated that the reattachment of the fractured fragment without any preparation of the coronal or root fragments results in lower bonding values [29, 30]. In this case, an internal dentinal groove was prepared on the coronal fragment to provide a higher mechanical strength and longevity.

To reinforce the cervical level of the reattached tooth, it is recommended to use an intracanal post since these interlock the coronal and root fragments and also minimize the stress on the reattached tooth fragment [31–33]. It has been suggested that the use of a long, thin fiber post is effective for reducing tensile stress that can lead to tooth root fractures of the anterior teeth with endodontic treatments [34]. In the present case, the fracture line was extending subgingivally and the tooth was pulpless. Hence, it was decided to gain intraradicular retention by using fiber posts.

## 4. Conclusion

Crown-root fractures localized in the anterior region need to be evaluated from several perspectives including tooth vitality, tissues involved, fracture location, and the quantity of remaining tooth structure. In the present case, multidisciplinary adhesive surgical approaches provided good results in terms of maintaining the tooth's structural integrity. Furthermore, a fragment reattachment of the tooth with a complicated crown-root fracture with an intracanal fiber post system proved to be clinically successful after treatment.
